# Supplementary material for: Acceptance and compliance with micronutrient powder (MNP) among children aged 6–23 months in northern Nigeria
Source: PLOS Glob Public Health. 2022 Oct 17;2(10):e0000961. doi: 10.1371/journal.pgph.0000961 (PMC10022258; doi:10.1371/journal.pgph.0000961)
Supplement: S1 Dataset — (ZIP) [file pgph.0000961.s008.zip › S8_Dataset/In-depth Interviews.zip!/In-depth Interviews/12072015_A_GU_PM_2_CG.pdf]

INTERVIEW WITH RAUTHA-A CAREGIVER IN LAKUMNA COMMUNITY IN GUYUK LOCAL GOVT.ADAMAWA STATE.

Q:DESCRIBE FOR ME THE PEOPLE IN YOUR IMMEDIATE FAMILY LIVING IN YOUR HOUSEHOLD?

R:We are seven in numbers.....7 female and one male.

Q:OF THIS 7 PEOPLE,HOW MANY OF THEM ARE AGES 6-24 MONTHS?

R:It is only one person and she is a female.

Q:WE HAVE HEARED OF FOOD CHALLENGES FOR YOUNG CHILDREN IN THIS SETTING RELATED TO THE SEASON....CAN YOU TELL ME THE COPING MECHANISM YOU DEVELOPE IN OTHER TO TACKLE THIS PROBLEM?

R:At such time, as a mother we can go into petty trading within the community, or be involved in farming activities for others for financial benefits(we work for them in their farms and they pay us instantly)

Q:HOW DOES THIS CHALLENGES AFFECT INFANT AND YOUNG CHILD FEEDING?

R:It will surely affect them, because once such times comes around everybody in the family bears the pain..... the adults in the family could understand the prevailing situation, but at least, for the kids, we can always try our best to provide for them because they are just kids and we have to fend for them no matter what the challenge is.

Q:ONE THING WE HAVE HEARD IN OTHER INTERVIEWS THAT CAREGIVERS OFTEN GIVE THIER CHILDREN IS 6-23 MONTHS IS TEA.....COULD YOU PLEASE EXPLAIN THE TERM TEA?

R:If you want to prepare tea for your child you need lipton, bournvita, milk and sugar. But you will have to ensure that the lipton is boiled in warm water for some minutes then you can now mix the other items to make a tea.

Q:DO YOU NORMALLY PREPARE THIS TEA FOR THIS YOUR 6 MONTHS BABY?

R:Never,she is too young for such, it is for other children like close to two years or above two years.

Q:TELL ME ABOUT YOUR EXPERIENCE WITH THE MNP POWDER THAT WAS GIVEN TO YOU SOMETIMES BACK?

R:Well,when i started to administer the mnp powder to my baby....i notice she was passing out a kind of blackish pooh-pooh.....i was afraid, but i still continue to give her the powder....then gradually her pooh-pooh returned back to normal.

Q:TELL ME ABOUT ANY POSITIVE ASPECT OF THIS MNP SO FAR?

R:Well...i cant really say but i noticed that the baby’s skin has changed and she has increased her eating volume, also i can notice that she has added a little weight....

Q:ASIDE THE EARLER CHALLENGED YOU TAKKED ABOUT..WAS THERE ANY OTHER CHALLENGED YOU ENCOUNTERD DURING THIS MNP TRIAL?

R:No there was none.

Q:SO HOW DID YOU HANDLE THE ISSUE OF THE BLACKISH POOH-POOH BY YOUR BABY?

R:I told you, i did nothing....i just continue to give her the mnp......until everything went back to normal.

Q:WERE THERE ANY BARRIRERS THAT MADE IT DIFFICULT FOR YOU TO APPROPRIATELY USE THE MNP?

R:To be honest with you, there was none.

Q:SOME CAREGIVERS MENTIONED THAT IT WAS HARD FOR THEM TO STORE THE MNP WITHOUT IT BEING TAKEN OR EAREN BY RODENTS.DID YOU HAD ANY SIMILAR EXPERIENCE?

R:No i have never for once had such experience....i have a particular place i normally keep this powder....it is only when i want to use it then i will bring it out and after usage i will return it back to the place....i don’t joke with things like this.......

Q:SOME MOTHERS INDICATED THAT IT MIGHT BE HARD TO REMEMBER TO USE THE MNP.DID YOU HAVE A SIMILAR EXPERIENCE......AND WHAT DID YOU DO TO REMEMBER?

R:No i have never for once forgotten to give the baby this powder....me am a different kind of mother, i no the importance of things like this.....i don’t think i can ever forget....

Q:SO WHAT DO YOU THINK CAN BE DONE TO ENCOURAGE OTHER MOTHERS TO BE LIKE YOU?

R:Well this depend on individual differences....some mothers don’t care about such things while others take it very seriously......the only thing is to properly sensitize and educate them on the importance and benefits of this mnp to their children.

Q:HOW DO YOU NORMALLY USE THIS MNP?

R;I usually use kunu to mix it with the mnp, if for example i prepare the kunu i will allow it to cool off, then i will fetch a little that would be sufficient for the baby, mix the mnp then give it to her to drink by myself.

Q:WHICH TIME DO YOU NORMALLY GIVE HER THE MNP?

R:Mostly, it is in the morning, after she has suckled breast.

Q:TO WHAT EXTENT DO YOU THINK YOUR BABY EITHER LIKES OR DISLIKE THE TASTE OF THIS MNP?

R:Well,she is still very small to determine properly.....but she always takes it anytime i give it t her...but sometimes she might refuse and i have to force it on her. i don’t think she normally refuse as a result of the taste......but you know little kids they have their own ways of behaviour....but generally she usually drinks the mnp mix with kunu.

Q:HAVE NOTICED ANY CHANGES IN HER EATING PATTERN SICE YOU STARTED TO USE THIS MNP?

R:Like i told you earlier on, her consumption has slightly increased.....she now drinks more kunu...unlike in the past......

Q:HAS THIS AFFECTED HER BREASTMILK CONSUMPTION?

R:No it has not.

Q:IN OTHER COMMUNITIES WE NOTICED THAT SHARING OF MNP OCCURED.COULD YOU TALKED ABOUT ANY SHARING WHEN YOU USED IT?

R:No such thing happened around here, i don't of the other women......but as for me i have never had such experience.

Q:WHAT IS THE REACTION OF YOUR HUSBAND GENERALLY ABOUT THIS MNP.....HAS HE EVER USED IT?

R:No he has never. The only he did when he came from the farm was to ask me about the mnp....who brought and what was the use of it.....i tried my little best to explain to him.....he now requested for the packs.....he studied it and gave it back to me.....that was all.

Q:DO OTHER FAMILY MEMBERS TRIES TO CONSUME THE LEFT OVER OF THIS MNP?

R:No. Not at all.

Q:SO WHAT DO YOU NORMALLY DO WITH THE LEFT-OVER OF THE MNP MIXED WITH KUNU?

R:It is very rare for the mnp to remain anytime i mixed it, because we were instructed to fetch a little quantity that the baby can consumed at a go......but occasionally you know little children they are unpredictable, if the mixed mnp is remained i normally throw it away

Q:DO YOU THINK 60 SATCHETS OF THIS MNP WILL BE ENOUGH FOR 60 DAYS.?

R:Since it is a dose per day....i think it should be enough.

Q:WHY DO YOU SAY SO....?

R:Well i think they that brought it no better.....if they had wanted us to use two or three per day they would have informed us.

Q:HOW DO YOU PERCEIVED THIS MNP.......DO YOU SEE IT AS A FOOD OR A DRUG OR SOMETHING ELSE....?

R:To me, i see it as a vitamin......i don't know if am right.....but that is how i view it.

Q:WHAT SORT OF VITAMIN?

R:A vitamin to give more vitality and strength.

Q:WHY DO YOU SAY SO?

R:No i don't have a particular reason......that is just how i perceived it.

Q:IF THIS MNP WERE TO BE SOLD IN THIS COMMUNITY VERY CHEAPLY WOULD YOU BUY IT?

R:I cant really speak for others because have personal choice and differences.....but as for me if i have the money i will buy it.....since i have tried it i assumed i know it's benefits to the baby.

Q:SO HOW MUCH DO YOU FEEL YOU CAN PAY FOR EACH SACHET?

R:I cant answer that question......what ever price you guys think will be good for local communities like ours.......is fine by me........but don't forget that this is a village and most people don't have cash.......

Q:NOW TELL ME SOME WAYS YOU BELIEVE CAN BE EFFECTIVE IN PROMOTING THIS MNP TO CAREGIVERS IN THIS COMMUNITY SO THAT THEY WOULD COMPLY WITH USING IT APPROPRIATELY?

R:There is no better ways than to mobilise the women and to lecture them on the benefits they stand to derive for their children should they use this mnp effectively.......like the lady that brought the mnp to my house gave a practical demonstration on how to use the mnp ....in short, she made prepare the kunu in her presence and she demonstrated how to apply the mnp......since then i have been following her instructions.......

Q:YOU MENTIONED MOBILISING WOMEN......WHERE DO YOU THINK WILL BE MORE APPROPRIATE?

R:I cant suggest that......may be if you meet the village head he may give you a better option.

Q:WHAT PEOPLE MOST INFLUENCE YOUR OPINIONS AND KNOWLEDGE ABOUT FEEDING PRACTICES?

R:I don't think i look up to anybody.....i try my best to handle my problem all alone....i don't being noticed too much....because am the shy and quiet type......

Q:SO IF YOU PROBLEMS WITH THIS YOUR BABY AM SEEING WHO DO YOU GO TO?

R:I simply pray to God.

Q:WHO INFLUENCES YOUR DECISION REGARDING FEEDING YOUR BABY THE MNP?

R:Well...the village head was very instrumental in making us to accept this powder and using it......because the day the lady brought this mnp.....it was at the village compound we gathered and were selected and given the powder......

Q;WHERE DO YOU THINK WILL BE VERY APPROPRIATE TO DISSEMINATE HEALTH NINFORMATION RELATED TO MNP?

R:Well......i don't really know.....but i feel the village head compound will be most appropriate.

Q:WHY DO YOU THINK SO?

R:He is like a father figure to everybody in this community.....people will easily listen to him when he gives an order or a directive......

Q:I STILL ASK YOU ONCE MORE.....WHO IN YOUR COMMUNITY DO YOU TRUST THE MOST ABOUT FOR RECEIVING MESSAGES ABOUT INFANT AND YOUNG CHILD FEEDING?

R:The community hospital.

Q:WHAT FORMS OF MEDIA DO YOU PREFER FOR RECEIVING MNP MESSAGES?

R:It is not about me alone.....but the entire community......if there is something that involves health....the town crier will be very appropriate to go round and inform the people.....may be they should assemble in the village head compound or in the community hospital.

Q:PERSONALLY......WHICH WILL YOU PREFER?

R:The hospital.

Q:WHAT DO YOU THINK OF RADIO OR SMS THROUGH PHONE?

R:Both will be good......but you have to consider how many women personally has a phone......or how many of us can effectively read.....if not to make simple call.....people do not do not use phones for any other thing around here.....

Q:WHAT ABOUT WORDOF MOUTH?AND BY WHOM

R:As you can see by yourself......this is a very small community....you can go round it in a very short time.....if you have any important message to pass all you need is to inform the town announcer to spread the news......or for him to tell the people to assemble at the chief compound or community hospital for an important announcement.......

Q:DO YOU HAVE A SPECIFIC EXAMPLE(S) OF OTHER SUCCESSFUL HEALTH EDUCATION CAMPAIGNS IN THIS COMMUNITY?

R:None that i can remember.....

Q:I WANT TO HEAR SOME SPECIFIC MESSAGES YOU THINK WILL BE MOST EFFECTIVE IN PROMTING THIS MNP?

R:Tell them something that will really touch them.....may be like telling them that this powder will cure some certain diseases, or it will enable their children to grow properly, or that the powder will develop the bones or skin of their children or it will make their children to be very intelligent......anything that will really make sense to the people.....see whether you like it or not...this is not a city....it is a village and people have their individual worries and problems....so you have to approach them with meaningful and beneficial information....something that is a bit different from what they are used to hearing......

Q:CAN YOU GIVE ME SOME EXAMPLES......

R:You know you are very funny.........are you not a doctor.....you should know better than i do.

Q:OK.....LETS CONTINUE....I AM NOT A DOCTOR ANYWAY.......SO WHAT CAN WE DO TO LIMIT THE SHARING OF THIS OF THIS MNP IN FAMILIES OR IN THE COMMUNITY AS WHOLE?

R:Educate the people.....re-educates them and continue to educates them the more....and if possible there should be periodic checks or visits by external people like you......because you know villagers really appreciates and respects people outside their territory.

Q:FINALLY,IS THERE ANYTHING ELSE YOU CAN SHARE ABOUT YOUR EXPERIENCE WITH THIS PRODUCT?

R:Well i feel the product is good and i can recommend it to anybody who seek my sincere advice...i have used it for the past one month and i believe it is something which is good for little baby's from two years below....and i know that the government will not introduce something they know will be detrimental to the well-being of little children or even their parents....though i have completed the dosage given to me but something tells me that the end will be good for both the baby and me her mother.

Q:ON A FINALE NOTE...... AND I AM REALLY SORRY FOR TAKEN MUCH OF YOUR TIME......IS THERE ANYTHING YOU CAN RECCOMEND TO US TO BE ABLE TO DEVELOPE AN EFFECTIVE MNP PROGRAMME?

R: If you really want to succeed in this your programme i suggest you carry along the village head in collaboration with women group leaders who can serve as focal persons in penetrating other women in the community.......alternatively you can involve the health workers since most of them are known and they too equally know the people.........i don't think if there can be a better way than this......though this is my own little understanding.....you can go round and ask other women about their opinion.

Q:THANK YOU VERY MUCH!!
